# Evaluating the strategies to control SARS-CoV-2 Delta variant spread in New Caledonia, a zero-COVID country until September 2021

**DOI:** 10.1016/j.ijregi.2023.06.004

**Published:** 2023-06-30

**Authors:** Noé Ochida, Myrielle Dupont-Rouzeyrol, Pierre-Henri Moury, Thibaut Demaneuf, Ann-Clair Gourinat, Sébastien Mabon, Marc Jouan, Simon Cauchemez, Morgan Mangeas

**Affiliations:** a UMR ENTROPIE, IRD, Université de La Réunion, IFREMER, Université de Nouvelle-Calédonie, CNRS, Noumea, New Caledonia; b Research and Expertise Unit on Dengue and Arboviruses, Institut Pasteur of New Caledonia, Pasteur Network, Noumea, New Caledonia; c Department of Anesthesia and Intensive Care Medicine, Grenoble University Hospital, Grenoble, France; d Research and Expertise Unit of Epidemiology, Institut Pasteur of New Caledonia, Pasteur Network, Noumea, New Caledonia; e Intensive Care Unit, Gaston-Bourret Territorial Hospital Center, Dumbea-Sur-Mer, New Caledonia; f Directorate of Health and Social Affairs, Noumea, New Caledonia; g Microbiology Laboratory, Gaston-Bourret Territorial Hospital Center, Dumbea-Sur-Mer, New Caledonia; h Mathematical Modelling of Infectious Diseases Unit, Institut Pasteur, Université Paris Cité, UMR2000, CNRS, Paris, France

**Keywords:** COVID-19, SARS-CoV-2, Zero-COVID, Non-pharmaceutical interventions, Vaccine, Modelling

## Abstract

**Objectives:**

New Caledonia, a former zero-COVID country, was confronted with a SARS-CoV-2 Delta variant outbreak in September 2021. We evaluate the relative contribution of vaccination, lockdown, and timing of interventions on healthcare burden.

**Methods:**

We developed an age-stratified mathematical model of SARS-CoV-2 transmission and vaccination calibrated for New Caledonia and evaluated three alternative scenarios.

**Results:**

High virus transmission early on was estimated, with *R*_0_ equal to 6.6 (95% confidence interval [6.4-6.7]). Lockdown reduced *R*_0_ by 73% (95% confidence interval [70-76%]). Easing the lockdown increased transmission (39% reduction of the initial *R*_0_); but we did not observe an epidemic rebound. This contrasts with the rebound in hospital admissions (+116% total hospital admissions) that would have been expected in the absence of an intensified vaccination campaign (76,220 people or 34% of the eligible population were first-dose vaccinated during 1 month of lockdown). A 15-day earlier lockdown would have led to a significant reduction in the magnitude of the epidemic (-53% total hospital admissions).

**Conclusion:**

The success of the response against the Delta variant epidemic in New Caledonia was due to an effective lockdown that provided additional time for people to vaccinate. Earlier lockdown would have greatly mitigated the magnitude of the epidemic.

## Highlights

• We modeled a COVID-19 outbreak and response in a zero-COVID Pacific territory.
• The SARS-CoV-2 Delta variant showed high initial transmission (R_0_ of 6.6).
• An effective lockdown resulted in a 73% reduction in transmission.
• Post-lockdown, the vaccination campaign prevented an epidemic rebound.
• An earlier lockdown could have substantially mitigated hospital load.

## Introduction

Totaling millions of deaths and even more hospitalizations, the COVID-19 pandemic has taken a terrible toll on global health [1]. Worldwide, public health authorities have been faced with the daunting responsibility of stopping or at least slowing down the transmission of the virus to best protect the population. At the start of the pandemic, initial national response strategies have been broadly categorized as elimination (also labeled zero-COVID) strategies i.e., maximum action to exclude disease and eliminate community transmission; or mitigation strategies i.e., “flatten the peak” to avoid overcrowding of health services [2,3]. To implement their response, countries have made extensive use of non-pharmaceutical interventions (NPIs), such as mandatory mask-wearing and physical distancing (including gathering bans), school, restaurant and non-essential shop closures, quarantine systems, stay-at-home orders, or curfews. Several of these measures were applied together under the term lockdown.

New Caledonia, a French Pacific Island territory, offers an interesting illustration of the zero-COVID approach. Upon the discovery of few imported (original strain) and local cases (Alpha variant), lockdowns were implemented for 1 month in March 2020 and March 2021. In both cases, the approach successfully eliminated the epidemic and the territory was declared COVID-19-free [4,5]. The lockdown consisted of the closure of all places of social life, a stay-at-home order for all persons not carrying out “essential work”, one authorized outing lasting at most 1 hour per day within a 1 km radius from home, and mandatory mask-wearing outside home. Once the lockdown was lifted, border closures for non-residents and mandatory quarantine on entry for residents were instituted, placing the territory in a so-called sanitary airlock. Apart from that, no particular control measures against COVID-19 were implemented; the whole territory's strategy was based on maintaining the integrity of the sanitary airlock from April 2020.

**Figure 1:**
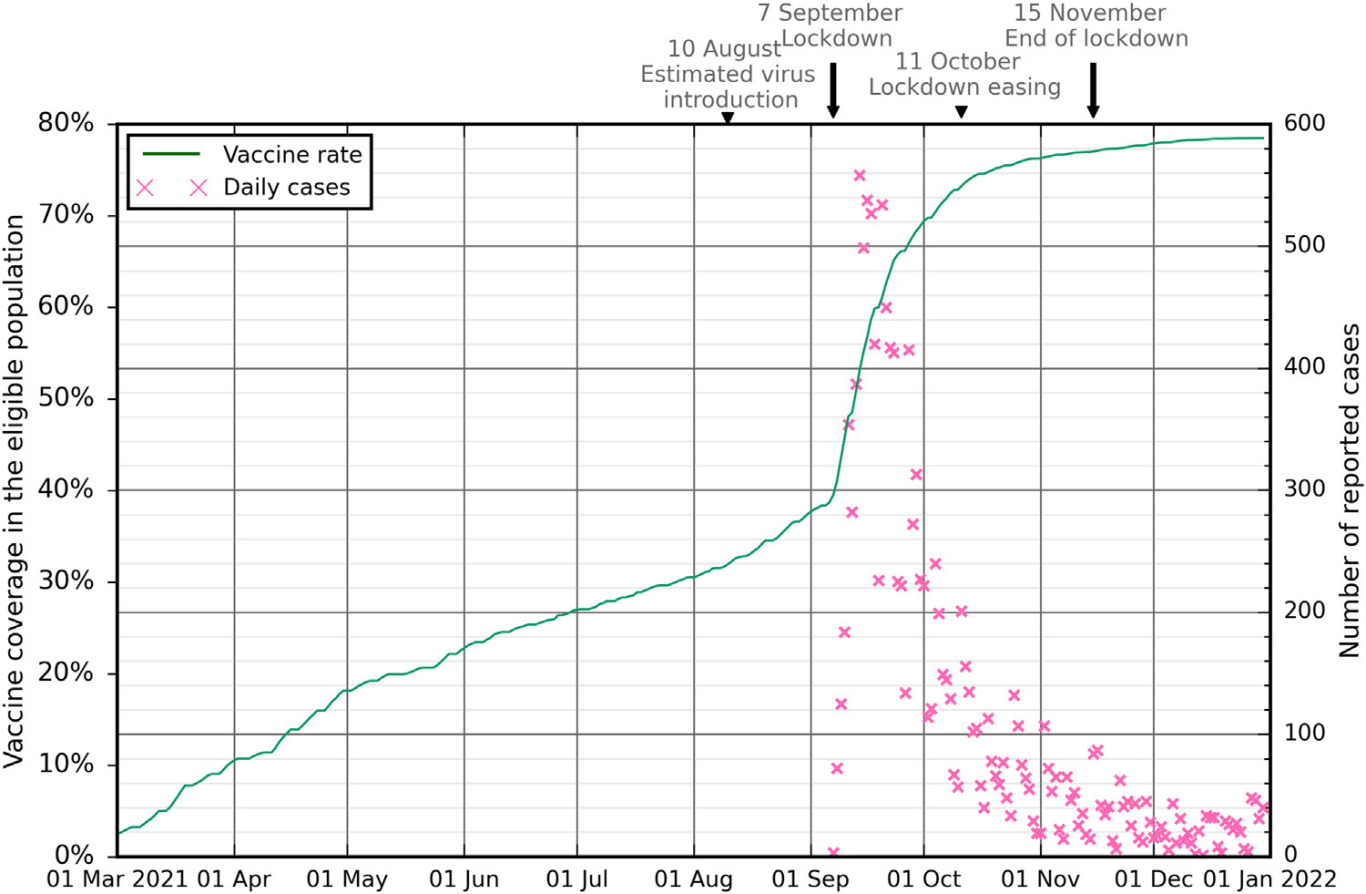
Daily reported COVID-19 cases, vaccine coverage, and interventions timeline in New Caledonia from March 2021 to January 2022. Daily COVID-19 reported cases (pink crosses) and vaccine coverage in the eligible population (i.e., ≥12 years old) (green solid line).

On September 06, 2021, a breach in the sanitary airlock was discovered when three unrelated cases admitted the previous days with severe pneumopathy (the first on September 3) tested positive for SARS-CoV-2 infection. A lockdown was announced that evening and implemented the next day, September 7. At that time, 39% of the eligible population had received at least one vaccine dose. In parallel, population screening was intensified, uncovering a large epidemic of locally transmitted cases (Figure 1). During the following week of September 13, hospital admissions escalated despite the lockdown with an average of 47 admissions per day. Thereafter, reported cases and daily hospital admissions steadily declined. During the 1-month lockdown period, from September 7 to October 10, 2021, 76,220 eligible people (34%) received their first vaccine dose over a total population of ∼271,000 inhabitants. New Caledonia authorities eased the lockdown on October 11 ending the stay-at-home order and resuming most work activities. This period after October 11 is known as the partial lockdown. During this period, an average of six hospital admissions per day was reported. After the partial lockdown ended no rebound was noted.

In this study, we report the experience of the COVID-19 epidemic due to the Delta variant in New Caledonia, South Pacific. Our objective was to assess the impact of the lockdown and the intensification of the vaccination campaign on the control of the epidemic. We also investigated how the timing of lockdown implementation affected the amplitude of the epidemic wave. This was achieved with a mathematical modeling of the epidemic that specifically described the impact on the health services system in terms of hospital admissions and intensive care unit (ICU) occupancy.

## Results

### Calibration and parameters estimation

**Figure 2:**
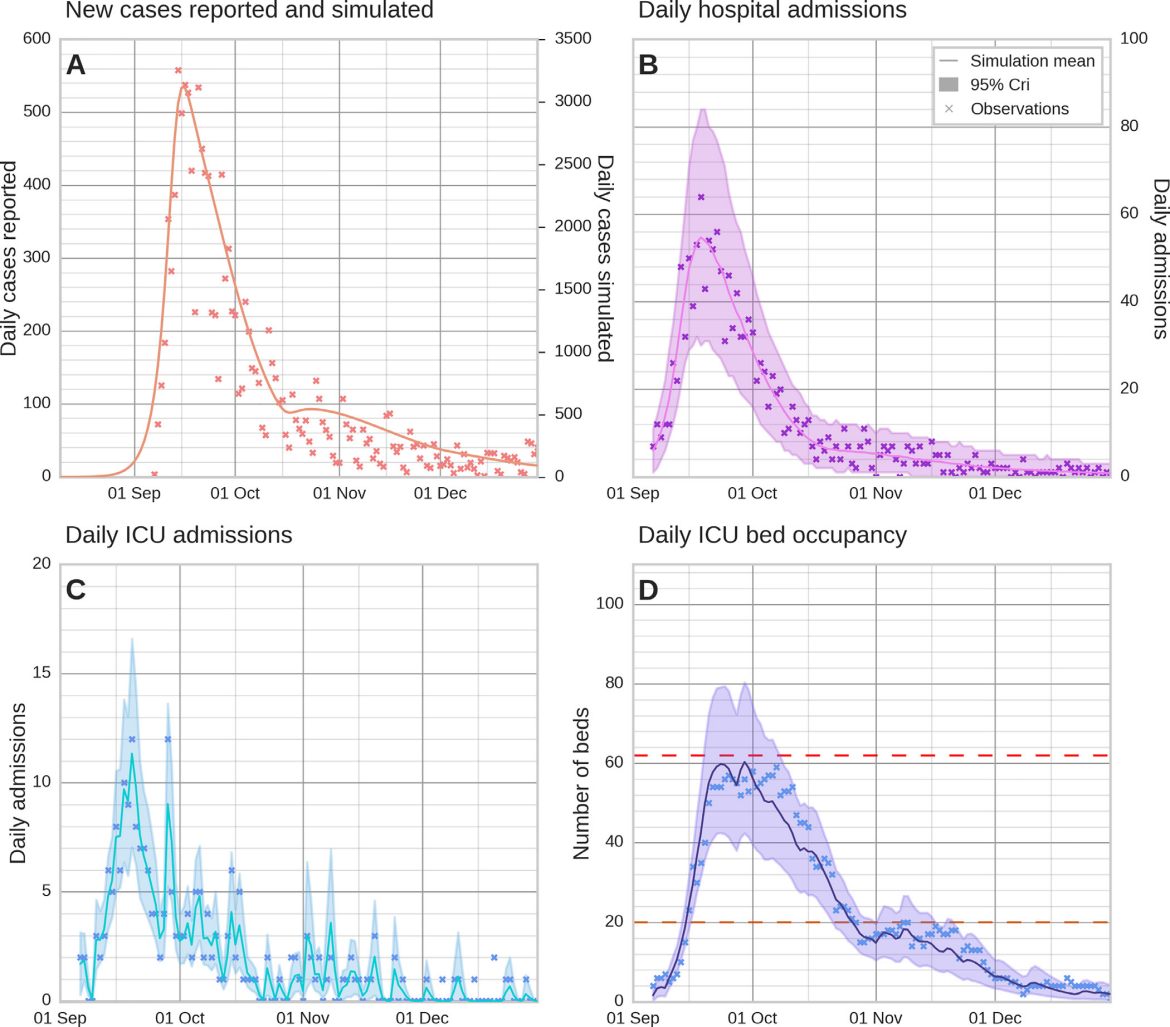
Comparison of the model simulations to observations. Panel A shows simulated daily infections, panel B shows simulated daily hospitalizations, panel C shows daily ICU admissions, and panel D shows the projected ICU bed occupancy; ICU dynamics have been simulated using daily ICU admission probabilities given hospitalization. Observational data points are marked with crosses. Solid lines and shaded areas represent the mean simulation and the 2.5^th^-97.5^th^ percentile range, respectively, from 1000 simulations using 1000 random samples from the posterior distribution of parameters estimated through Markov Chain Monte Carlo sampling. Dashed lines represent the maximum number of ICU beds under normal (orange) and COVID (red) regimes. ICU: intensive care unit.

The model satisfyingly described the dynamics of daily hospital admissions (Figure 2b), which was our calibration target. Using daily probability of ICU admission given hospitalization estimates, we were able to statistically reconstruct ICU admissions (Figure 2c) and ICU bed occupancy (Figure 2d). The model simulation shows that the daily cases peaked at approximately 3000 around September 15^th^, a week after the lockdown was implemented (Figure 2a). Details regarding infections and hospitalization in each age group can be found in Supplementary Figure 1.

Table 1 summarizes our estimates for the basic reproduction number $R_{0}$ and the impact of the successive interventions relative to the pre-lockdown $R_{0}$. Pre-lockdown, $R_{0}$ was estimated at 6.6 (95% confidence interval [CI: 6.4-6.7]). We estimated that the implementation of the lockdown dropped the $R_{0}$ to 1.8; 73% reduction (95% CI [70-76%]). After the lockdown, during the next period of partial lockdown i.e., when most work activities resumed and the stay-at-home order was kept only during weekends, the $R_{0}$ increased to 4.0; a 39% reduction compared to pre-lockdown $R_{0}$. Ending the partial lockdown, i.e., ending the stay-at-home orders on the weekends, had little effect on $R_{0}$, it increased to 4.5. Estimated/calibrated parameters are presented in Supplementary Table 1.

**Table 1:** Estimates of the basic reproduction number $R_{0}$ at different periods.

Period	Measures	$R_{0}$	95% confidence interval	Reduction
Pre-lockdown	No measure.	6.6	6.4-6.7	Baseline
Lockdown	School closures, closing of stores except for the first necessity stores, obligation to remain at home for “non-essential” workers, 1 hour's outing per day within a 1 km radius of the domicile with a mandatory mask.	1.8	1.5-2.0	73%
Partial lockdown	Work resumes during the week and stay-at-home order on weekends, re-opening of schools and stores with a gauge, curfew from 10 pm to 5 am, family gatherings of up to 10 people authorized on weekdays, vaccine pass in restaurants.	4.0	3.5-4.6	39%
End of lockdown	Curfew from 10 pm to 5 am, family gatherings of up to 30 people authorized on weekdays, vaccine pass in restaurants.	4.5	3.9-5.1	32%

### Alternative scenarios

We then examined counterfactual modeling scenarios describing how the trajectory of this epidemic would have changed if different interventions had been implemented. In these scenarios, we used the average probability of ICU admission given hospitalization (estimated over the epidemic) to reconstruct ICU dynamics, rather than using daily probabilities as in Figure 2. The impact on ICU dynamics of using daily versus average probabilities of ICU admission given hospitalization is shown in Supplementary Figure 2. The goal was to circumvent the modeling of context-specific ICU admissions practices, as they are not well generalizable to alternative scenarios (refers to Discussion).

**Figure 3:**
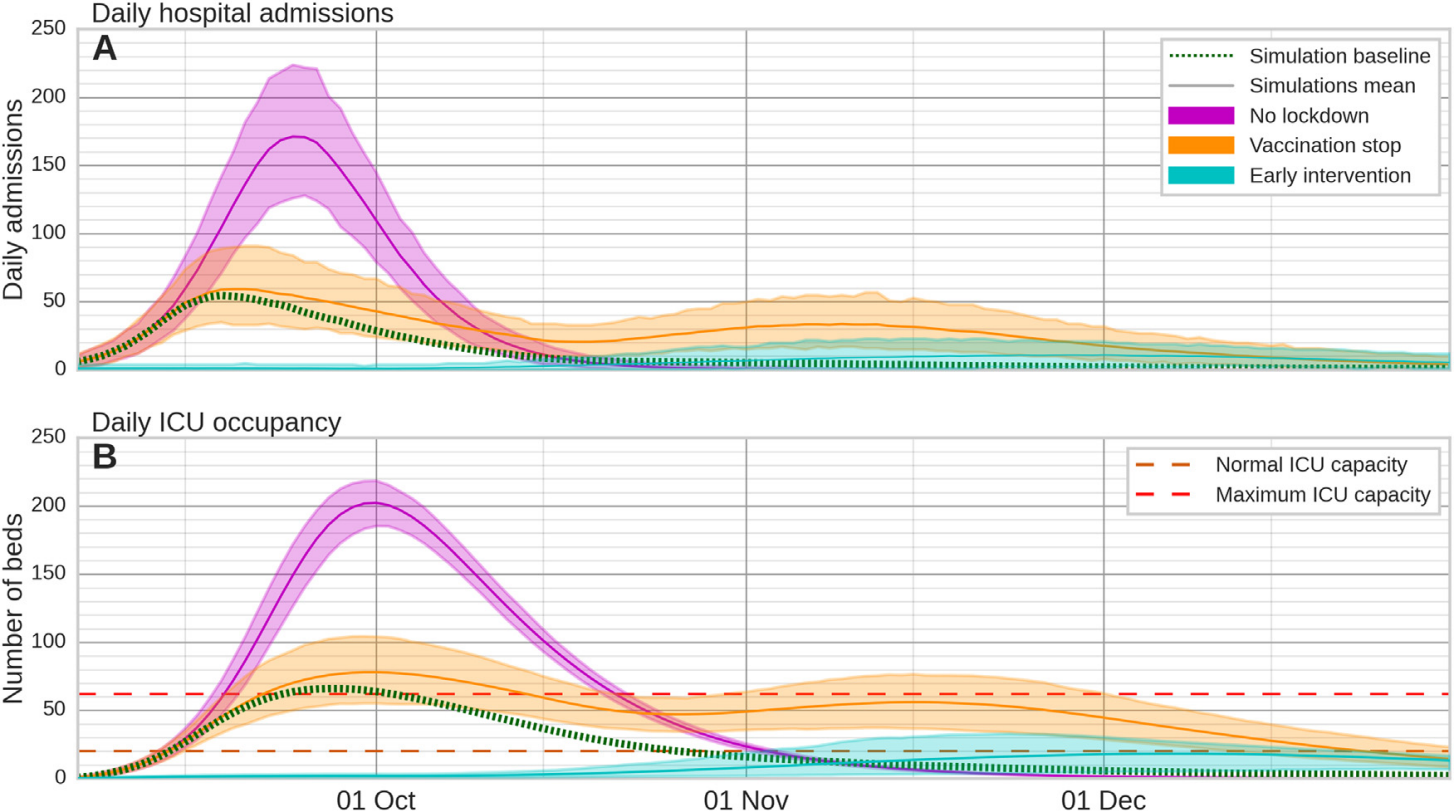
Alternative scenarios compared to observations of hospital admission and ICU bed occupancy. Panel A shows simulated daily hospital admissions and panel B shows daily ICU bed occupancy (simulated using the average probability of ICU admission given hospitalization over the entire epidemic). Scenario A: purple, no lockdown is implemented; Scenario B: orange, no vaccination after lockdown; Scenario C: blue, 2 weeks earlier implementation of the lockdown and associated measures. The mean baseline simulation (no scenario) is represented by a dotted green line. Solid lines and shaded areas represent the mean simulation and the 2.5^th^-97.5^th^ percentile range, respectively, from 1000 simulations using 1000 random samples from the posterior distribution of parameters estimated via Markov Chain Monte Carlo sampling. Dashed lines represent the maximum number of ICU beds under normal (orange) and COVID (red) regimes. ICU: intensive care unit.

If no lockdown (nor other non-pharmaceutical measures) had been implemented (scenario A), twice as many hospital admissions would have been expected and the peak of ICU bed occupancy would have been three times higher than that observed, a catastrophic scenario (Figure 3, Table 2). Note that in this scenario, we make the assumption that people would have not reduced their contacts (refers to Discussion). If no vaccines had been administered from the beginning of the lockdown to the end of the simulation (scenario B), we would have expected an epidemic rebound at the easing of the lockdown (October 11) and twice as many hospital admissions as in our baseline scenario. The peak in ICU bed occupancy would have been of the same order as in baseline. Lastly, if there had been early detection of the virus achieved through comprehensive surveillance, enabling the timely implementation of measures such as a lockdown initiated 2 weeks earlier (scenario C), it would have significantly curtailed the epidemic and delayed hospital admissions until the easing of the lockdown restrictions. Hospital admissions would have been cut by half and, at the peak, ICU bed occupancy would have remained well below maximum capacity.

**Table 2:** Estimates of hospital admissions and ICU occupancy according to three different scenarios.

	Total number of hospital admissions	Peak ICU bed occupancy
Baseline	1415	66
Scenario A	3087 (+118%)	202 (+206%)
Scenario B	3053 (+116%)	78 (+18%)
Scenario C	663 (-53%)	18 (-73%)

## Discussion

In this study, we characterized the Delta variant epidemic in New Caledonia. Using mathematical modeling and alternative scenarios, we estimated the relative contribution of the lockdown and vaccination on the control of the epidemic. We also assessed how the timing of the lockdown impacted healthcare burden.

Results showed that before the lockdown, there was high viral transmission with $R_{0}$ around 6.6. This value places New Caledonia in the high range of Liu et al.’s review of five estimates of the Delta variant in different provinces of China and UK (mean; 5.08; range 3.2-8) [6]. Contrary to these locations, the epidemic of the Delta variant in New Caledonia was the first active circulation of SARS-CoV-2 in the territory and went initially unnoticed by the surveillance network and therefore by the population. Epidemiological investigations estimated that viral introduction most likely occurred on August 10, 2021. Thus SARS-CoV-2 circulated in New Caledonia for 1 month before its discovery. Then, the implementation of the lockdown resulted in a 73% reduction of $R_{0}$. Our estimates are consistent with those obtained for lockdowns implemented in Europe during the first pandemic wave in spring 2020 [7], [8], [9]. NPIs implemented during the lockdown in New Caledonia included school and workplace closures and restrictions on gatherings (private or public); these interventions have already been identified as effective in other countries [6,[10], [11], [12], [13], [14]]. Our results provide further evidence of the effectiveness of NPIs to reduce SARS-CoV-2 spread.

After the lockdown was initiated, hospital admissions declined rapidly, contrary to our alternative scenario A where no lockdown is implemented. This scenario results in twice as many hospital admissions and three times the peak occupancy of ICU beds. A catastrophic scenario where the majority of patients would have suffered from severely degraded quality of care likely resulting in high in hospital mortality. In reality, as hospital admissions declined rapidly, the lockdown was eased into a partial lockdown after 1 month. The easing of the lockdown did not result in an epidemic rebound, in contrast to our model prediction for the alternative scenario B, characterized by no vaccination campaign after the lockdown. Scenario A and B highlight the beneficial interaction between the vaccination campaign and the effective reduction of contacts due to the lockdown in New Caledonia. Finally, scenario C involves implementing the lockdown 2 weeks earlier. In this scenario, the epidemic is mostly under control, highlighting the beneficial effects of early detection and response, as already shown elsewhere [15,16]. Scenario C is a strong argument for strengthening surveillance systems, particularly in the context of Pacific Island Countries and Territories (PICTs) [17].

In the alternative scenarios, the probability of ICU admission given hospitalization ($p_{ICU}$) was set by using the average probability over the entire epidemic. In reality, the probability of ICU admission varied over time because it depended on physician input. Indeed, we saw a decrease in the estimated probability of ICU admission as the ICU approached its maximum bed occupancy capacity (Supplementary Figure 3). Concurrently, it was also a time of high hospital admissions (Supplementary Figure 4). In the post-peak phase of hospital admissions, a period characterized by reduced hospital admissions, we observe that the daily estimates of the probability of ICU admission given hospitalization, are more irregular, contingent on patient arrival (Supplementary Figure 4). Epidemiological context was different than the pre-peak period; the pressure on ICU services lessened, enabling a more flexible admission policy in the ICU. Additionally, the demographic profile of the patients may have also evolved, potentially encompassing late arrivals at the hospital. As the variations of daily ICU admission probabilities depend on the magnitude of the epidemic, i.e., number of hospital admissions and the resources of the hospital at a given time, the daily ICU estimates that are context-dependent are not well generalizable for the alternative scenarios. This is why $p_{ICU}$ has been set at the average over the whole period in the alternative scenarios. For reference, Supplementary Figure 5 shows the alternative scenarios using the estimated daily $p_{ICU}$. In Supplementary Figure 2, we see that when using the average $p_{ICU}$, the peak in daily ICU bed occupancy estimates exceeds the observations and in particular the total ICU bed capacity. It is a reminder that the capacity ceiling for intensive care beds was reached during this epidemic and that the ICU admission regime was disrupted as a result.

A possible limitation in this study involves scenario A in which no lockdown is implemented. It is very likely that the size of the epidemic is overestimated because this scenario assumes that people would not have reduced their social contacts. Presumably, in the event of a large-scale epidemic with increased hospitalizations and deaths, people would have changed their behavior. However, this scenario is worth considering because it informs us that the drastic decrease in social contacts due to the lockdown gave individuals time to vaccinate themselves and thus be protected against the severe effects caused by SARS-CoV-2 infection. A final possible limitation is that we did not model transmission heterogeneities that emerge from socio-demographic differences between individuals, such as age, employment, or ethnicity. Concerning age, in the absence of data, we assume homogeneous contacts among age groups. We refrain from using a specific age contact matrix, although such matrices have been used in COVID-19 modeling studies [7,11,12,16,18,19]. However, we assume that intergenerational contacts are rather homogeneous in New Caledonia because of the Oceanian lifestyle. Because vaccination and hospitalization risk are explicitly age-dependent in the model, we hope that this may help mitigate this bias. New Caledonia as a PICTs has a multicultural population composed of Melanesian, Polynesian, Asian, and European communities; for future research, it would be interesting to study existing heterogeneities in intra and inter-community contact networks or in access to healthcare. Eventually this social context could be integrated in epidemiological models leading to more tailored public health decisions [20,21]. A population-based prevalence survey of COVID-19 and/or an analysis of hospitalizations could be a first step in studying the burden of COVID-19 in different communities.

In this study, we showed that in New Caledonia, the lockdown greatly reduced SARS-CoV-2 transmission providing an additional delay for a large part of the population to get vaccinated and acquire a level of protection against the virus. The combination of lockdown and intensification of the vaccination complemented each other in a powerful response strategy that slowed and contained the Delta variant epidemic. We also showed that if the measures had been implemented 2 weeks earlier, the epidemic would have been mostly under control. Like other PICTs such as New Zealand, Wallis and Futuna, or Vanuatu, New Caledonia was able to close its borders quickly and thus act on the control of intra-territorial transmission of SARS-CoV-2. This insular context, the lockdown and the intensification of the vaccination campaign made it possible, despite a very strong pressure on the health services, to avoid more severe alternative scenarios.

## Material & methods

### Model description

Based on Bosetti et al. [18], we developed a deterministic aged-structured compartmental model of SARS-CoV-2 transmission and vaccination within the Susceptible-Exposed-Infectious-Recovered (SEIR) framework. The model divides the population into seven epidemiological compartments; susceptible (S), pre-infectious (E1), presymptomatic (E2), mildly ill (Imild), severely ill requiring hospitalization (Ihosp), hospitalized (H). These compartments are further divided into four vaccination status and six age groups 0-11, 12-15, 16-49, 50-64, 65-74, and 75+ years old. New Caledonia's population by age group is based on the 2019 census [22]. Individuals transition between compartments at rates that are inverse of assumed epidemiological durations. We assume a 4-day mean incubation period ($E_{1}\to E_{2}$) where individuals are infected but not infectious, followed by a 1-day mean presymptomatic infectious period ($E_{2}\to I_{mild}/I_{hosp}$). Additionally, we assume a 3-day symptomatic infection duration, after which individuals either recover or are hospitalized if it is a severe case ($I_{mild}\to R/I_{hosp}\to H$). The four vaccination status are based on a two-dose vaccination scheme: unvaccinated (no subscript), first-dose administered ($X_{V0}$), first level of vaccine protection ($X_{V1}$), and second level of vaccine protection ($X_{V2}$). A diagram of the model is presented in Supplementary Figure 6. Daily vaccinations by age group in New Caledonia, as recorded by the Directorate of Health and Social Affairs of New Caledonia, are used to populate the first-dose administered compartment. We assumed that individuals in the first-dose administered compartment (no protection yet) acquire the first level of vaccine protection after a mean period of 14 days (i.e., $X_{V0}\to X_{V1}$) and individuals in 1^st^ level of protection compartments acquire a second level of protection after a mean period of 14 days ($X_{V1}\to X_{V2}$). Almost all vaccination in New Caledonia has been with the Pfizer-BioNTech COVID-19 vaccine BNT162b2, as it was with a few exceptions, the only vaccine distributed in New Caledonia. The vaccine protection effects on susceptibility (i.e., the probability of contracting the virus upon a contact with an infectious individual), infectivity (i.e., the probability of transmitting the virus upon a contact with a susceptible individual) and severity (i.e., the probability of hospitalization if infected) are presented in Supplementary Table 2. We assumed that infection or vaccination conferred lasting immunity during the simulation period.

The probabilities of hospitalization following infection are influenced by age and the number of comorbidities. These probabilities presented in Supplementary Table 3, were derived from data on the prevalence of comorbidities by age, as well as the probabilities of hospitalization given SARS-CoV-2 infection according to age and the number of comorbidities in the French population, as presented by Kiem et al. [23]. We assumed that the prevalence of comorbidities was comparable between New Caledonia and France in the 18-74 years old (Supplementary Table 5). We additionally assume no comorbidities for individuals aged 0-17 and the same prevalence of comorbidities in the 75+ age group as in the 65-74 age group in France. Using probabilities of hospitalization given infection based on age and the number of comorbidities (Supplementary Table 6), the presumed comorbidity prevalence in the population of New Caledonia, and data from the 2019 New Caledonia census [22], we calculated a weighted average of the probabilities of hospitalisation given infection for the age groups considered in our model {0-11, 12-15, 16-49, 50-64, 65-74, 75+}. In calculating the averages, we assumed that the probabilities of hospitalization in age groups 0-11, 12-15, 16-29 in New Caledonia would be equivalent to those if age groups 0-9, 10-17, 18-29 in France respectively. Finally, we adjusted these probabilities to take account of the increased severity of the Delta variant, assuming a 50% increase in all age groups except for the 75+ [18]. Considering studies that indicate a lower susceptibility of children and adolescents to SARS-CoV-2 infection, we assumed that children under 12 years old and adolescents under 16 years old were 50% and 25% less susceptible to contracting the disease respectively [24].

### Estimation of epidemiological parameters and modeling the impact of interventions

We modeled the effect of implementing and easing the lockdown on the basic reproduction number $R_{0}$ and the effective reproduction number $R_{i}$ of each period i sequentially using a logistic function $f_{i}$ as follow:

$R_{i}\left(t\right)=R_{i-1}\times f_{i}\left(t\right)$ and $f_{i}\left(t\right)=1\pm \frac{\eta_{i}}{1+e^{-(t-t_{i+1}-\nu_{i})}}$. The sign of the second term in $f_{i}$ depends on whether it is a reduction (lockdown) or an increase (lockdown easing). $\eta_{i}$ is the either the reduction or increase rate parameter depending on the sign of the second term of $f_{i}$ and $\nu_{i}$ is the time when the decrease/increase is 50% effective. Given $t_{0}$ as the start of the simulation, $t_{1}$ as the date of the virus introduction, $t_{2}$ as the date of the lockdown, $t_{3}$ as the partial lift date, $t_{4}$ as the full lift date and $t_{5}$ as the end of simulation date, we first calculate $R_{1}$ from $t_{1}$ to $t_{3}$ as $R_{1}\left(t\right)=R_{0}\times f_{1}\left(t\right)$. Then using $R_{1}$, we calculate $R_{2}$ from $t_{1}$ to $t_{4}$ as $R_{2}\left(t\right)=R_{1}\times f_{2}\left(t\right)$ and using $R_{2}$, we calculate $R_{3}$ from $t_{1}$ to $t_{5}$ as $R_{3}\left(t\right)=R_{2}\times f_{3}\left(t\right)$. $R_{3}$ which spans from the start of the virus introduction to the end, accounts for all successive decreases or increases in the reproduction number. Thus, it is the effective reproduction number R(t) for the entire simulation.

Estimates of initial $R_{0}$, $\eta_{1}$ the reduction rate parameter and $\nu_{1}$ the time when lockdown is 50% effective, has been determined in a Bayesian framework. Assuming that hospital admissions followed a Negative Binomial process, posterior distributions of the parameters were estimated by Markov Chain Monte Carlo (MCMC) sampling. To update previous knowledge, we used a likelihood function using observed hospitalization admissions from the Gaston-Bourret Territorial Hospital Center (CHT), the only tertiary hospital in New Caledonia. $\alpha_{hosp}$ was an additional parameter, the overdispersion parameter of the Negative Binomial process. Then, to avoid compounding uncertainties when estimating the subsequent parameters involved in the logistic increase of $R_{1}$; $\left\{\left\{\eta_{2,}\nu_{2}\right\},\left\{\eta_{3,}\nu_{3}\right\}\right\}$, we used a least squares approach. The prior and posterior distributions of parameters for the initial period, as well as subsequent parameters estimated using a least squares approach, are presented in Supplementary Table 1. The 95% CI in our simulations are based on the 2.5^th^ and 97.5^th^ percentiles from 1000 random samples in the posterior distributions of parameters estimated via the Bayesian approach. MCMC sampling was implemented with the *PyMC* module and least squares with the S*cipy* module in Python 3.8.

### Statistical modeling of ICU admissions

To model ICU admissions and ICU occupancy from simulated hospital admissions, we first estimated the daily probabilities of ICU admission given hospitalization using observed hospital and ICU admissions data and $\tau_{Hosp-ICU}$, the mean delay time from hospital admission to ICU admission.

ws is a vector of length n+1 that holds the probabilities of an individual, who will ultimately be admitted to the ICU, entering ICU on a given day *i*, from day 0 to day *n*. Each element of *ws* is calculated as:

$\begin{array}{c} ws_{i}=F\left(x_{2}\right)-F\left(x_{1}\right) \end{array}$, $x_{1}=\left\{ \begin{array}{c} 0,\;\mathrm{if}\;i\;=\;0\\ i\;-\;0.5,\;\mathrm{otherwise} \end{array}\right.\;x_{2}=i+0.5$ and i∈{0,1,2,3,...,n−1,n}. F is the cumulative distribution function of the exponential distribution parametrized with $\lambda =1/\tau_{hosp-ICU}$.

ds is a vector which holds the daily count of individuals admitted to the ICU under the assumption that every hospital admission results in an ICU admission. Each element of ds is calculated as:

$ds_{i}=\sum_{j=0}^{i}\mathrm{adHos}p_{\mathrm{obs},j}\times ws_{i-j}$, i∈{0,1,2,3,...,n−1,n} and $adHosp_{obs}$ the time series of hospital admissions.

If $adICU_{obs}$ is the observed time series of ICU admissions, then the probability of ICU admission given hospitalization for a given day i is expressed as $pICU_{i}=\frac{adICU_{obs,i}}{ds_{i}}$. The actual observed number of ICU admissions divided by the total ICU admissions possible.

Finally given observed or simulated hospital admissions, daily ICU admissions are given by:

$$\mathrm{adIC}U_{i}=\sum_{j=0}^{i}\mathrm{adHos}p_{j}\times \mathrm{pIC}U_{j}\times ws_{i-j}$$

For the alternative scenarios simulations, instead of daily probabilities of ICU admission given hospitalization, we used average probability of ICU admission given hospitalization over the entire epidemic, $\mu_{P_{ICU}}$, this was calculated as the total sum of ICU admissions divided by the total sum of hospital admissions (Supplementary Table 4).

### Statistical modeling of ICU bed occupancy

$n_{s}$ is a vector of length n+1 that holds the probabilities that an individual admitted to the ICU will depart day i. Each element of $n_{s}$ is calculated as:

$ns_{i}=1-G\left(x\right)$, $x=\left\{ \begin{array}{c} 0.5,\;\text{if}\;i\;=\;0\\ i,\;\mathrm{otherwise} \end{array}\right.$. G is the cumulative distribution function of the exponential distribution parametrized with $\lambda =1/\tau_{ICU-R}$ and $\tau_{ICU-R}$ is the mean duration of ICU occupancy.

ICU occupancy is calculated as follow $\mathrm{occIC}U_{i}=\sum_{j=0}^{i}\mathrm{adIC}U_{j}\times ns_{i-j}$.

$\tau_{Hosp-ICU}$ and $\tau_{ICU-R}$ were obtained from anonymized data on the course of patients admitted to ICU.

### Assessment of three alternative scenarios

To assess the relative contribution of vaccination, lockdown and lockdown implementation time, three alternative scenarios were settled and analyzed.

Scenario A explores the contribution of the lockdown in the reduction of transmission of SARS-CoV-2, i.e., $R_{0}$. In this scenario $R_{0}$ remains at its pre-lockdown value from the date of the virus introduction until the end of the simulation.

Scenario B explores the contribution of vaccination to controlling the epidemic. In this scenario, there is no vaccination in population from lockdown until the end of the simulation.

Scenario C investigates how the epidemic trajectory would have been modified due to an earlier intervention. In this this scenario, we maintain the same durations for the lockdown and partial lockdown but shift their starting and ending dates 2 weeks earlier.

## Declaration of Competing Interests

The authors have no competing interests to declare.

## Appendix Supplementary materials

Image, application 1
