# Supplementary material for: Evaluating the strategies to control SARS-CoV-2 Delta variant spread in New Caledonia, a zero-COVID country until September 2021
Source: IJID Reg. 2023 Jun 30;8:64–70. doi: 10.1016/j.ijregi.2023.06.004 (PMC10423666; doi:10.1016/j.ijregi.2023.06.004)
Supplement: Supplementary file 1 [file mmc1.docx]

Supplementary material

Table of Contents

[Supplementary Figure 1. Simulated infections and hospitalizations by age and vaccination status. 2](#__RefHeading___Toc211066_4257398721)

[Supplementary Table 1. Estimated or calibrated parameters. 3](#__RefHeading___Toc41919_4101015160)

[Supplementary Figure 2. Comparison of simulated daily ICU admissions and bed occupancy using either daily or average 4](#__RefHeading___Toc41921_4101015160)

[Supplementary Figure 3. Estimates of daily probability of ICU admission given hospitalization and observations of ICU bed occupancy 5](#__RefHeading___Toc41923_4101015160)

[Supplementary Figure 4. Estimates of daily probability of ICU admission given hospitalization and observations of hospital admission 6](#__RefHeading___Toc41925_4101015160)

[Supplementary Figure 5. Alternative scenarios compared to observations of hospital admission and ICU bed occupancy. 7](#__RefHeading___Toc41927_4101015160)

[Supplementary Figure 6. Unstratified model structure 8](#__RefHeading___Toc41929_4101015160)

[Supplementary Table 2. Pfizer-BioNtech vaccine COVID-19 vaccine efficacy against SARS-CoV-2 Delta variant 10](#__RefHeading___Toc41931_4101015160)

[Supplementary Table 3: Hospitalisation probabilities for Delta variant infection across considered age groups in New Caledonia. 11](#__RefHeading___Toc41933_4101015160)

[Supplementary Table 4. Estimates of parameters involved in the statistical modelling of ICU admissions and bed occupancy. 12](#__RefHeading___Toc41935_4101015160)

[Supplementary Table 5. Prevalence of comorbidities across age groups in the French population aged 18 -74 years. 13](#__RefHeading___Toc41937_4101015160)

[Supplementary Table 6: Probabilities of hospitalization given infection by age and comorbidity for the Alpha variant infection in the French population aged 18 to 80+. 15](#__RefHeading___Toc41939_4101015160)

[References 16](#__RefHeading___Toc211769_4257398721)

##
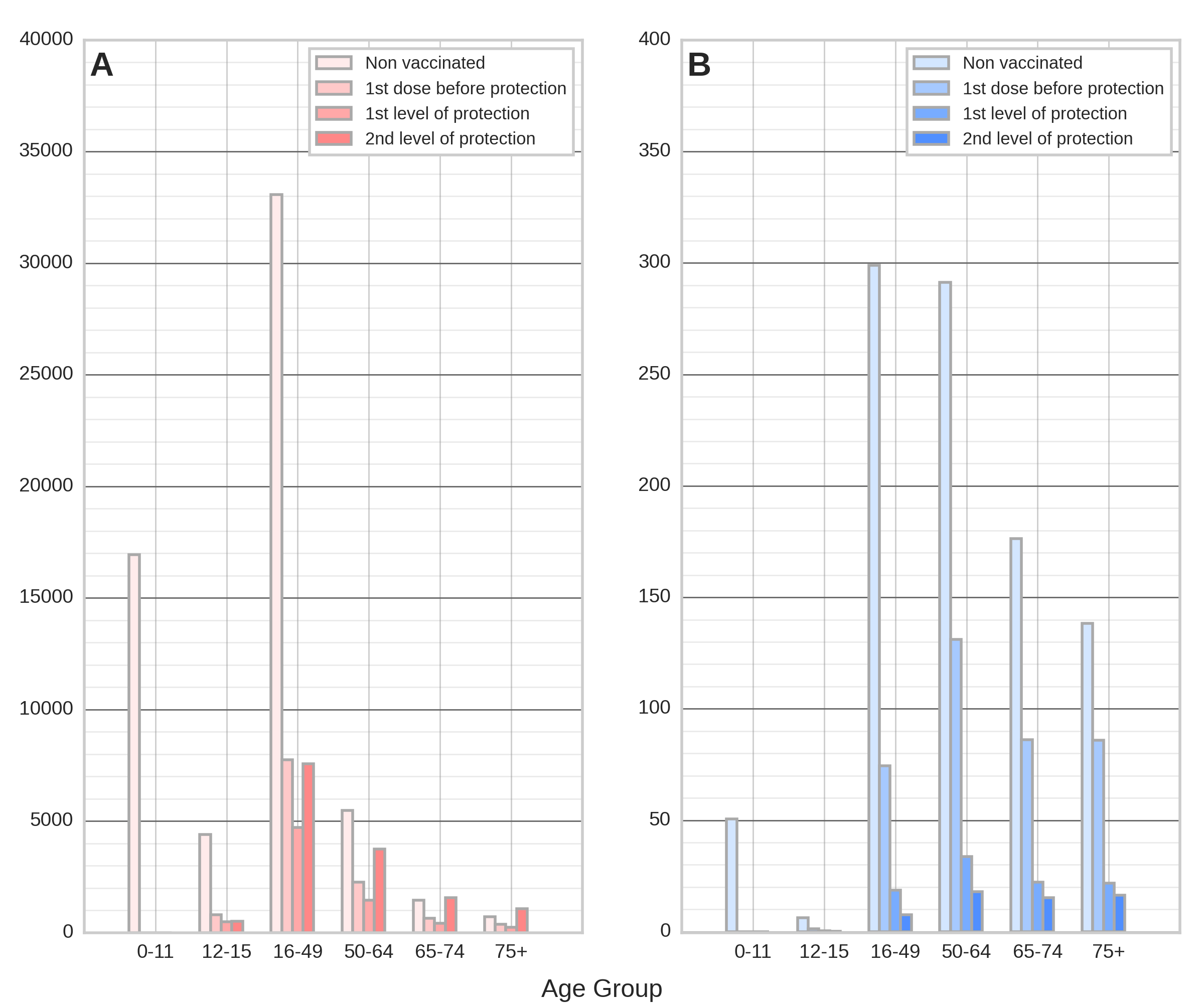


# **Supplementary Figure 1. Simulated infections and hospitalizations by age and vaccination status.**

A: Infections. B: Hospitalisations. The status "1st dose before protection" refers to individuals who received their first dose but have not yet developed protection against the virus.

In this figure, we can clearly see the unbalanced contribution between the number of infections and the number of hospitalizations based on an individual's age. However, we note a significant number of hospitalisation among 0-11 year old, who are three times more numerous than the 12-15 year old and are considered twice as likely to be hospitalized if infected (refer to Supplementary Text 1 and Supplementary Table 3).

| Parameter | Distribution | Prior distribution | Mean | 2.5^th^ – 97.5^th^ centile |
| --- | --- | --- | --- | --- |
| $R_{0}$ | Truncated normal | Mean: 6.00  Standard deviation: 2.00  Support : [1, 12] | 6.6 | 6.4 – 6.7 |
| $\eta_{1}$ | Beta | $\alpha=8$  $\beta=2$ | 0.73 | 0.71-0.76 |
| $\nu_{1}$ | Truncated exponential | $\lambda=\frac{1}{5}$  support [1, 15] | 5.0 | 3.7-6.3 |
| $\alpha_{hosp}$ | Exponential | $\lambda=5$ | 0.0127 | 5E-4 – 3.97E-2 |
| $\eta_{2}$ | - | - | 1.28 | - |
| $\nu_{2}$ | - | - | 4.3 | - |
| $\eta_{3}$ | - | - | 0.1 | - |
| $\nu_{3}$ | - | - | 15.0 | - |

# **Supplementary Table 1. Estimated or calibrated parameters.**

$R_{0}$,$\eta_{1}$,$\nu_{1}$,$\alpha_{hosp}$have been estimated via MCMC sampling in a Bayesian framework.

$\eta_{2}$, $\nu_{2}$, $\eta_{3}$, $\nu_{3}$ have been calibrated via least squares approach (Refers to Methods)

#
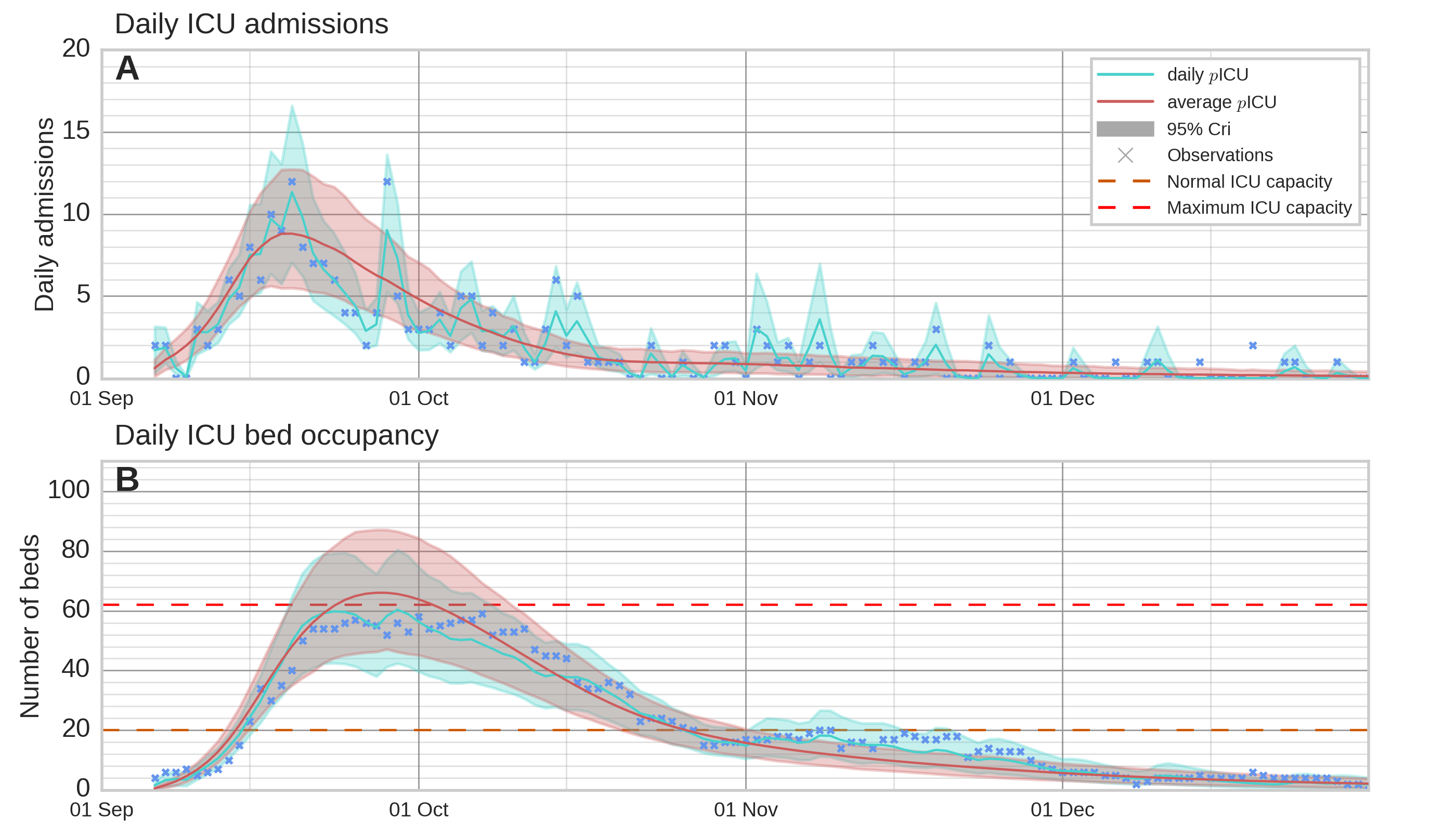


# **Supplementary Figure 2. Comparison of simulated daily ICU admissions and bed occupancy using either daily or average** $p_{ICU}$

Panel A shows simulated daily ICU admissions and the panel B represents daily ICU bed occupancy. $p_{ICU}$ is the statistical estimates of probability of ICU admission given hospitalisation. Blue lines and shaded areas represents simulated ICU dynamics using daily $p_{ICU}$ and red lines and shaded areas indicate simulated ICU dynamics using average$p_{ICU}$. The solid lines and shaded areas represent the mean simulation and the 2.5th – 97.5th percentile range, respectively, from 1000 simulations using 1000 random samples from the posterior distribution of parameters estimated via MCMC sampling. Dashed lines represent the maximum number of ICU beds under normal (orange) and COVID (red) regimes.

#
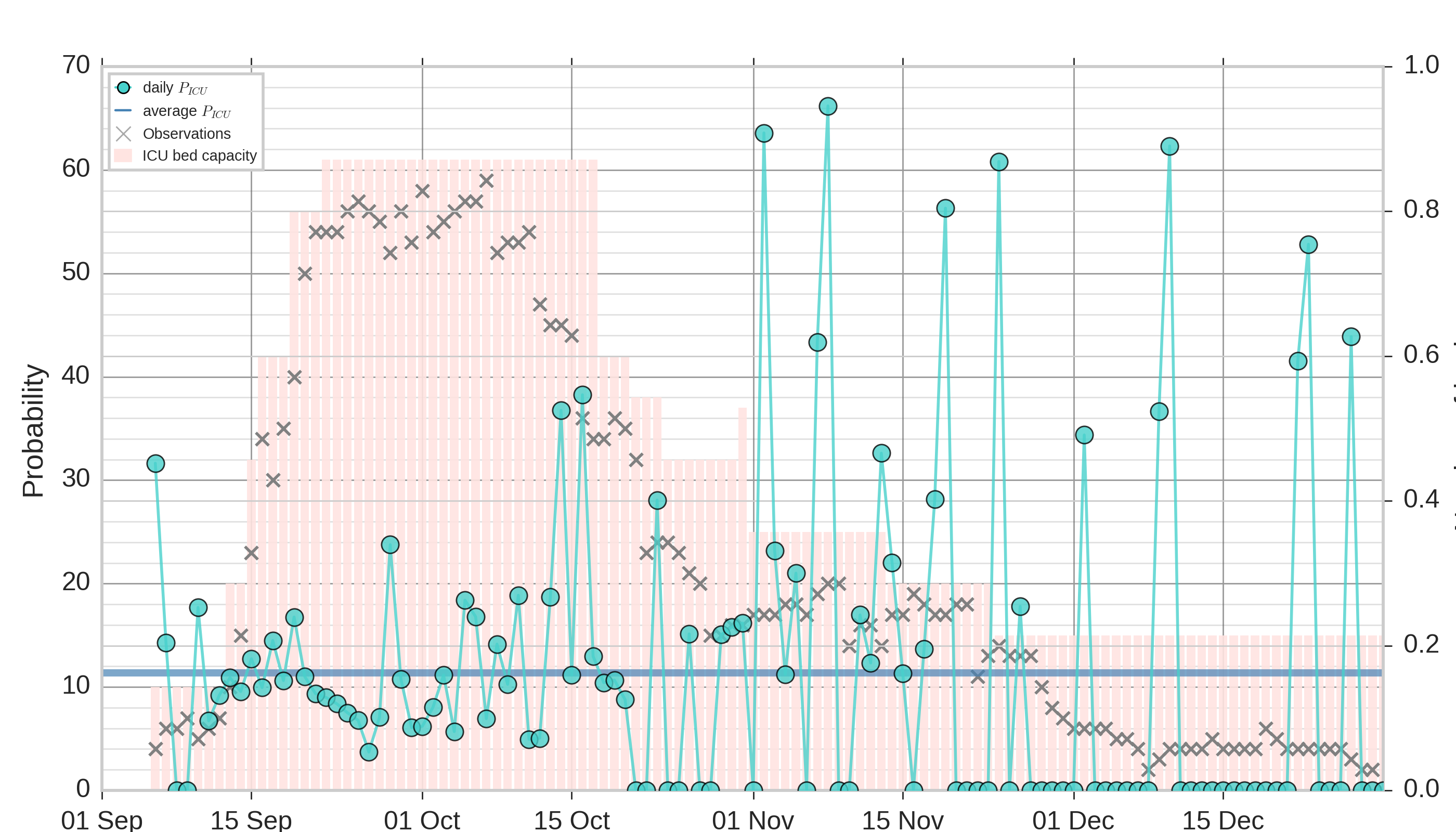


# **Supplementary Figure 3. Estimates of daily probability of ICU admission given hospitalization and observations of ICU bed occupancy**

Daily probabilities of ICU admission given hospitalization (blue marker and line), Observations of ICU bed occupancy (grey crosses), average probability of ICU admission given hospitalization (solid blue line), ICU beds capacity (pinks bars).

We observe a decrease in the probability of ICU admission at the peak of ICU bed capacity, observable around September 20.


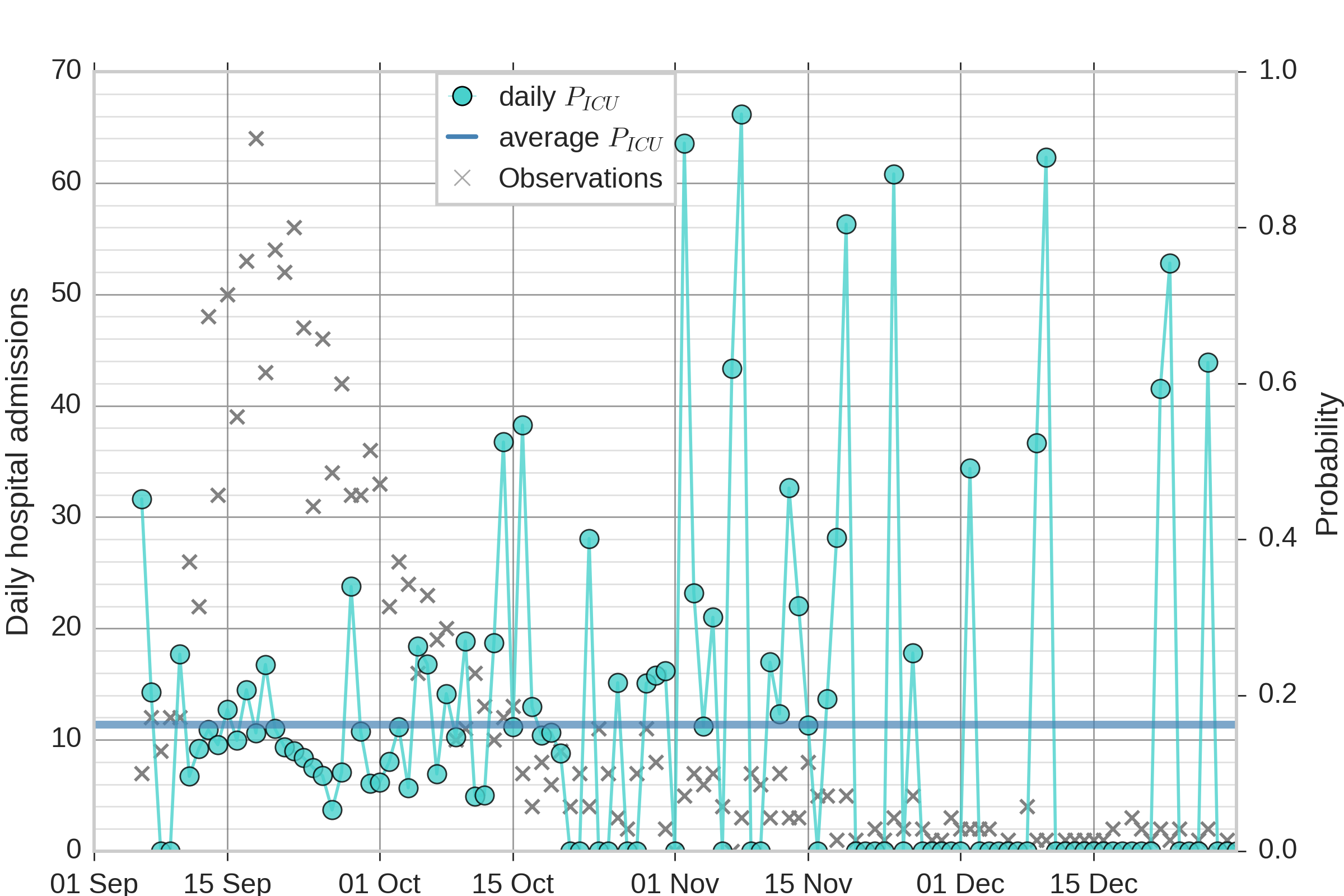


# **Supplementary Figure 4. Estimates of daily probability of ICU admission given hospitalization and observations of hospital admission**

Daily probabilities of ICU admission given hospitalization (blue marker and line), Observations of hospital admission (grey crosses), average probability of ICU admission given hospitalization (blue line).

Post-peak hospital admission, a period of diminished hospital admission, we see that the estimates of daily probability of ICU admission given hospitalisation are more irregular and saw-toothed

#
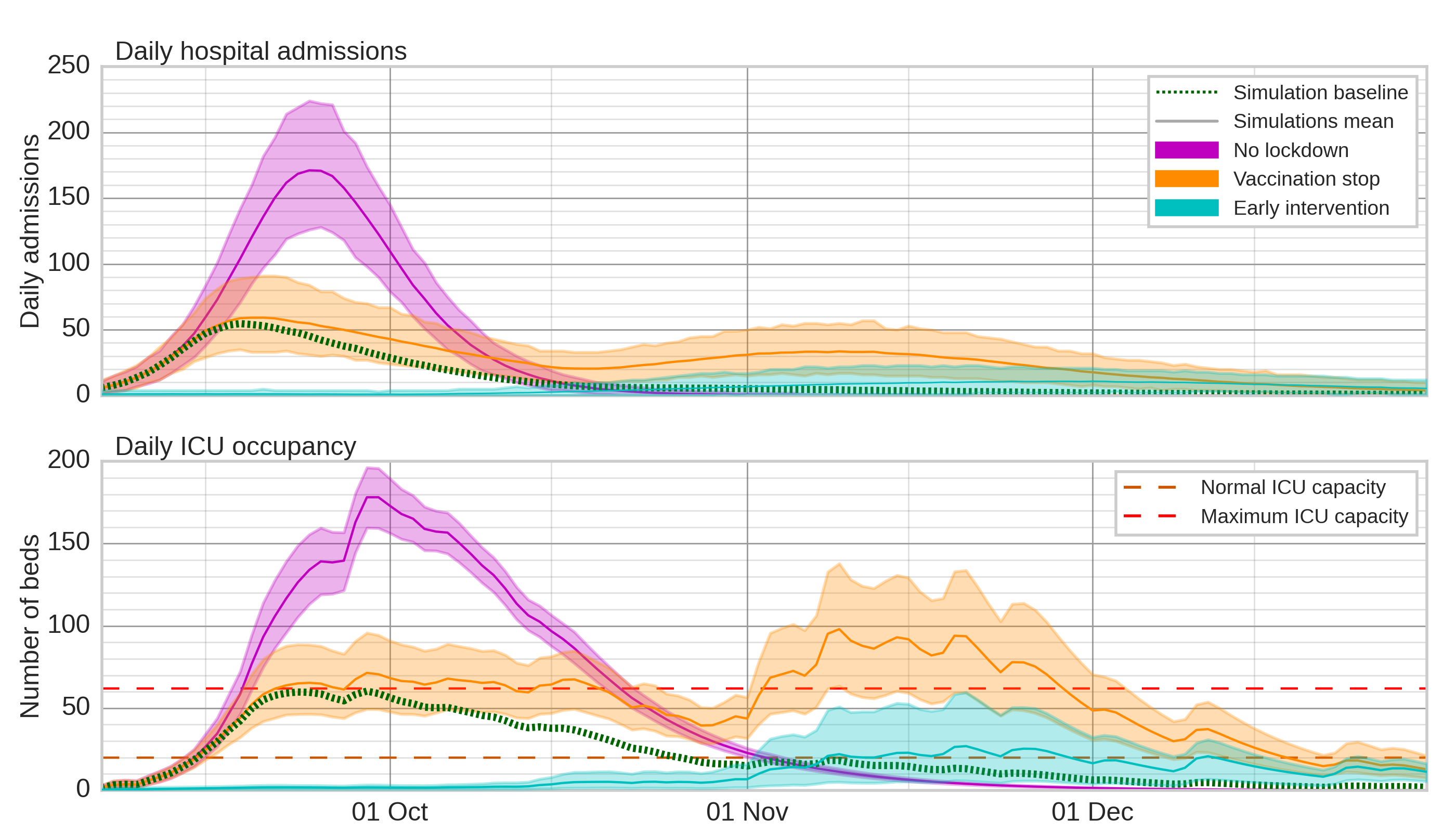


# **Supplementary Figure 5. Alternative scenarios compared to observations of hospital admission and ICU bed occupancy.**

Panel A shows simulated daily hospital admissions and panel B shows daily ICU bed occupancy (simulated using daily probabilities of ICU admission given hospitalization). Scenario A: purple, no lockdown is implemented ; Scenario B: orange, no vaccination after lockdown ; Scenario C: blue, two weeks earlier implementation of the lockdown and associated measures. The mean baseline simulation (no scenario) is represented by a dotted green line. Solid lines and shaded areas represent the mean simulation and the 2.5th – 97.5th percentile range, respectively, from 1000 simulations using 1000 random samples from the posterior distribution of parameters estimated via MCMC sampling. Dashed lines represent the maximum number of ICU beds under normal (orange) and COVID (red) regimes.


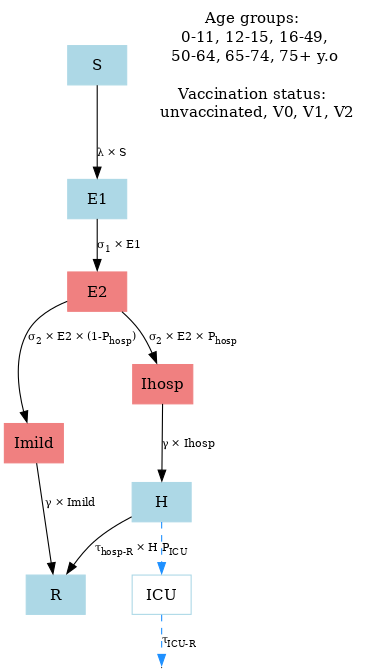


# **Supplementary Figure 6. Unstratified model structure**

$S$: susceptible , $E1$: pre-infectious , $E2$: pre-symptomatic:, $Imild$: mildly ill , $Ihosp$: severely ill requiring hospitalization,$H$: hospitalized : and $R$ recovered : Solid line arrows with Greek letters represent transition rate between compartments in the SEIR model. $\sigma_{1}=\frac{1}{4}$; $\sigma_{2}=1$; $\gamma=\frac{1}{3}$ . Dashed arrows represent the statistical modelling framework of ICU dynamics with $P_{ICU}$ the probability of ICU admission given hospitalisation and $\tau_{ICU-R}$ the mean duration of ICU bed occupancy. $\lambda$ is the force of infection and is defined as $\lambda=\frac{\beta}{N}\sum_{j=1}^{4} \left( 1-\alpha_{inf}\left( j \right) \right)\sum_{i=1}^{6} E_{2ij}+I_{mildij}+I_{hospij}$. $\beta$ is the transmission parameter defined as $\beta=R_{0}\left( \sigma_{1}+\gamma\right)$, where $R_{0}$ is the basic reproduction number. $N$ is the total population in the model. $\alpha_{inf}\left( j \right)=V_{E,infectivity}\times1_{\left\{ 3,4 \right\}}\left( j \right)$ with $V_{E,infectivity}$ the vaccine protection against the probability of transmitting the virus upon a contact with a susceptible individual, $j$ the vaccination status, $j=1$: unvaccinated, $j=2$: $V_{0}$, $j=3$: $V_{1}$, $j=4$: $V_{2}$. $1_{\left\{ 3,4 \right\}}$is the indicator function, it equals to 1 if $j$ equals to 3 or 4, 0 otherwise.

$i$ subscripts represent age groups. Individual in blue compartments are non infectious and in red compartments infectious.

|  |  | Protection |
| --- | --- | --- |
| Effectiveness against severe disease (severity) | one dose | 85% |
|  | two dose | 95% |
| Effectiveness against mild disease or infection (susceptibility) | one dose | 33% |
|  | two dose | 85% |
| Effectiveness against infectiousness if infected (infection) | one and two dose | 40% |

# **Supplementary Table 2. Pfizer-BioNtech vaccine COVID-19 vaccine efficacy against SARS-CoV-2 Delta variant**

This table has been adapted from Sonabend et al., 2021 [1].

| **Age group** | **Probability of hospitalisation** |
| --- | --- |
| 0-11 | 0.003 |
| 12-15 | 0.0015 |
| 16-49 | 0.0095 |
| 50-64 | 0.0582 |
| 65-74 | 0.1335 |
| 75+ | 0.2183 |

# **Supplementary Table 3: Hospitalisation probabilities for Delta variant infection across considered age groups in New Caledonia.**

| **Parameter** | **Meaning** | **Estimation** |
| --- | --- | --- |
| $\mu_{P_{ICU}}$ | Average probability of ICU admission given hospitalisation | 0.16 |
| $\tau_{Hosp-ICU}$ | Mean duration between hospital arrival and ICU admission | 0.5 days |
| $\tau_{ICU-R}$ | Mean duration of ICU bed occupancy | 10.6 days |

# **Supplementary Table 4. Estimates of parameters involved in the statistical modelling of ICU admissions and bed occupancy.**

| Number of comorbidities | 18-49 | 95% CI | 50-64 | 95% CI | 65-74 | 95% CI |
| --- | --- | --- | --- | --- | --- | --- |
| 0 | 84.3 | (81.0-87.4) | 69.3 | (64.8–73.9) | 58.3 | (51.7-65.0) |
| 1 | 14.2 | (11.1-17.3) | 19.2 | (15.4-23.0) | 20.2 | (15.0-25.4) |
| 2 | 1.3 | (0.4-2.3) | 6.2 | (3.8-8.5) | 13.2 | 13.2(7.9-18.5) |
| 3+ | 0.2 | (0.00-0.5) | 5.3 | (3.0-7.7) | 8.3 | 8.3(4.5-12.1) |

# **Supplementary Table 5. Prevalence of comorbidities across age groups in the French population aged 18 -74 years.**

This table adapted from Kiem et al. 2020[2], illustrates the prevalence of comorbidities across age groups aged 18 -74 years. To ensure that the total percentage equals 100%, minor adjustments of ±0.1% have been made to the original values in the grey cells.

|  |  |  |
| --- | --- | --- |
| **Age** | **Number of comorbidities** | **P(H\|I)** |
| 0-9 | 0 | 0.002 |
|  | 1 | 0.005 |
|  | 2 | 0.011 |
|  | 3 + | 0.011 |
|  |  |  |
| 10-17 | 0 | 0.001 |
|  | 1 | 0.002 |
|  | 2 | 0.004 |
|  | 3 + | 0.005 |
|  |  |  |
| 18-29 | 0 | 0.003 |
|  | 1 | 0.008 |
|  | 2 | 0.018 |
|  | 3 + | 0.019 |
|  |  |  |
| 30-39 | 0 | 0.004 |
|  | 1 | 0.012 |
|  | 2 | 0.026 |
|  | 3 + | 0.028 |
|  |  |  |
| 40-44 | 0 | 0.006 |
|  | 1 | 0.016 |
|  | 2 | 0.035 |
|  | 3 + | 0.037 |
|  |  |  |
| 45-49 | 0 | 0.012 |
|  | 1 | 0.012 |
|  | 2 | 0.034 |
|  | 3 + | 0.063 |
|  |  |  |
| 50-54 | 0 | 0.021 |
|  | 1 | 0.021 |
|  | 2 | 0.06 |
|  | 3 + | 0.112 |
|  |  |  |
| 55-59 | 0 | 0.029 |
|  | 1 | 0.029 |
|  | 2 | 0.084 |
|  | 3 + | 0.157 |
|  |  |  |
| 60-64 | 0 | 0.04 |
|  | 1 | 0.04 |
|  | 2 | 0.116 |
|  | 3 + | 0.216 |
|  |  |  |
| 65-69 | 0 | 0.052 |
|  | 1 | 0.052 |
|  | 2 | 0.052 |
|  | 3 + | 0.2 |
|  |  |  |
| 70-74 | 0 | 0.096 |
|  | 1 | 0.096 |
|  | 2 | 0.096 |
|  | 3 + | 0.37 |
|  |  |  |
| 75-79 | 0 | 0.144 |
|  | 1 | 0.144 |
|  | 2 | 0.144 |
|  | 3 + | 0.554 |
|  |  |  |
| 80+ | 0 | 0.206 |
|  | 1 | 0.206 |
|  | 2 | 0.206 |
|  | 3 + | 0.794 |

# **Supplementary Table 6: Probabilities of hospitalization given infection by age and comorbidity for the Alpha variant infection in the French population aged 18 to 80+.**

Derived from Kiem et al., 2020 [2], this table presents the probabilities of hospitalization given an Alpha variant infection, categorized by age group and comorbidity status in the French population.

# References

[1] Sonabend R, Whittles LK, Imai N, Perez-Guzman PN, Knock ES, Rawson T, et al. Non-pharmaceutical interventions, vaccination, and the SARS-CoV-2 delta variant in England: a mathematical modelling study. The Lancet. 2021 Nov;398(10313):1825–35. <https://doi.org/10.1016/S0140-6736(21)02276-5>.

[2] Kiem CT, Massonnaud C, Levy-Bruhl D, Poletto C, Colizza V, Bosetti P, et al. Evaluation des stratégies vaccinales COVID-19 avec un modèle mathématique populationnel [Internet] [document]. Haute Autorité de Santé ; Institut Pasteur Paris ; Santé publique France; 2020 [cited 2022 Jun 27]. Available from: <https://hal-pasteur.archives-ouvertes.fr/pasteur-03087143>
